# Platelet-rich plasma preparation for regenerative medicine: optimization and quantification of cytokines and growth factors

**DOI:** 10.1186/scrt218

**Published:** 2013-06-07

**Authors:** Paola Romina Amable, Rosana Bizon Vieira Carias, Marcus Vinicius Telles Teixeira, Ítalo da Cruz Pacheco, Ronaldo José Farias Corrêa do Amaral, José Mauro Granjeiro, Radovan Borojevic

**Affiliations:** 1 Excellion Biomedical Services, Petrópolis, Rio de Janeiro, Brazil; 2 Post-graduation Program of Morphological Sciences, Institute of Biomedical Sciences, Federal University of Rio de Janeiro, Rio, RJ, Brazil; 3 Bioengineering, National Institute of Metrology, Quality and Technology, Rio, RJ, Brazil

**Keywords:** Platelet-rich plasma, Centrifugation, Growth factors, Cytokine, Activation

## Abstract

**Introduction:**

Platelet-rich plasma (PRP) is nowadays widely applied in different clinical scenarios, such as orthopedics, ophthalmology and healing therapies, as a growth factor pool for improving tissue regeneration. Studies into its clinical efficiency are not conclusive and one of the main reasons for this is that different PRP preparations are used, eliciting different responses that cannot be compared. Platelet quantification and the growth factor content definition must be defined in order to understand molecular mechanisms behind PRP regenerative strength. Standardization of PRP preparations is thus urgently needed.

**Methods:**

PRP was prepared by centrifugation varying the relative centrifugal force, temperature, and time. Having quantified platelet recovery and yield, the two-step procedure that rendered the highest output was chosen and further analyzed. Cytokine content was determined in different fractions obtained throughout the whole centrifugation procedure.

**Results:**

Our method showed reproducibility when applied to different blood donors. We recovered 46.9 to 69.5% of total initial platelets and the procedure resulted in a 5.4-fold to 7.3-fold increase in platelet concentration (1.4 × 10^6^ to 1.9 × 10^6^ platelets/μl). Platelets were highly purified, because only <0.3% from the initial red blood cells and leukocytes was present in the final PRP preparation. We also quantified growth factors, cytokines and chemokines secreted by the concentrated platelets after activation with calcium and calcium/thrombin. High concentrations of platelet-derived growth factor, endothelial growth factor and transforming growth factor (TGF) were secreted, together with the anti-inflammatory and proinflammatory cytokines interleukin (IL)-4, IL-8, IL-13, IL-17, tumor necrosis factor (TNF)-α and interferon (IFN)-α. No cytokines were secreted before platelet activation. TGF-β3 and IFNγ were not detected in any studied fraction. Clots obtained after platelet coagulation retained a high concentration of several growth factors, including platelet-derived growth factor and TGF.

**Conclusions:**

Our study resulted in a consistent PRP preparation method that yielded a cytokine and growth factor pool from different donors with high reproducibility. These findings support the use of PRP in therapies aiming for tissue regeneration, and its content characterization will allow us to understand and improve the clinical outcomes.

## Introduction

The major functions of platelets are preventing acute blood loss and repairing vascular walls and adjacent tissues after injury. During wound healing, platelets are activated by contact with collagen, exposed to the bloodstream after endothelial injury. Platelets secrete stored intercellular mediators and cytokines from the cytoplasmic pool and release their α-granule content after aggregation. This secretion is intense in the first hour and platelets continue synthesizing more cytokines and growth factors from their mRNA reserves for at least another 7 days [1]. More than 800 different proteins are secreted into the surrounding media [1,2], having a paracrine effect on different cell types: myocytes [3], tendon cells [3-7], mesenchymal stem cells from different origins [8-11], chondrocytes [12-14], osteoblasts [3,15,16], fibroblasts [17-19] and endothelial cells [20]. Cell proliferation, angiogenesis and cell migration are stimulated, resulting in tissue regeneration. There are also reports confirming that platelets secrete antimicrobial peptides, suggesting an antibiotic effect [21].

Other properties were already proven for platelets related to their anti-inflammatory and analgesic effects [22-24]. A clinical trial showed that platelet concentrates had an analgesic effect [25] and Asfaha and colleagues showed PAR_4_-mediated analgesic effects *in vitro*[26]. El-Sharkawy and colleagues studied platelet secretions and their effect on macrophage cultures, concluding that platelet concentrates function as an anti-inflammatory agent, because of the high RANTES and LXA4 concentrations [27].

Platelet-derived products include platelet-rich plasma (PRP), which can be used with or without previous platelet activation. Such preparations have been used since the 1970s and they have been increasingly popular since the 1990s [28]. Since then, different ways of preparing PRP have emerged: from conventional blood centrifugation to commercial systems; activated by adding collagen, calcium and/or thrombin, by glass contact or by freezing cycles; applied as platelet suspension or as a gel; and the methodology continues to broaden [29-31].

The application of PRP in different tissues has given promising results in different pathologies such as acute and chronic injuries of bone and cartilage. Kon and colleagues reported observation of 91 patients (115 knees) treated with PRP, which showed that PRP treatment is safe, reduces pain and improves knee function, especially in younger patients at 12 months [32]. This was superior to hyaluronic acid viscosupplementation [32]. Subsequent analysis at 24 months, however, showed a progressive loss of the improvement and opened up the question of possible repeated therapies [33]. This example indicates the necessity for further studies even in series with an extensive number of patients and provided with controls. General opinion in recent reviews is that the majority of reported clinical studies do not have sufficient statistical power to give conclusive results. In view of multiple potential PRP applications in orthopedics, sports medicine and reparative surgery, comparative analyses of different clinical scenarios would be useful. These comparisons are not feasible, mainly because PRP is a biological product, prepared using different protocols, sometimes without even controlling whether platelets were effectively concentrated and purified or whether an early activation occurred, discarding all of the secreted growth factors within the platelet-poor plasma (PPP). Another issue that is not even mentioned in clinical reports is whether there is a correlation between the platelet concentration or the PRP volume applied per injured area or volume. Studies have already demonstrated that low platelet concentration is inefficient and that high concentrations have an inhibitory effect on cell growth, but results are still contradictory [34-36]. Although still not deeply characterized, the leukocyte content was also shown to be an important factor, increasing inflammation and reducing tissue regeneration in tendinopathies [37]. Preparation procedures are also relevant, as shown by studies of the chondro-inductive and osteo-inductive potential of PRP, which is reported to be lost by thrombin activation [38] and retained after freeze–thaw activation [39]. Consistent nomenclatures, standardized protocols to produce PRP as well as a full characterization of the final product are still missing and would highly improve the comparability of studies [40,41].

Combinations of PRP and mesenchymal stem cells have been widely studied *in vitro*[8-11]. All of the authors concluded that PRP increased cell proliferation but divergences were found regarding the stem cell differentiation capacity, some concluding that PRP favored the osteogenic differentiation [9] and others demonstrating a chondrogenic compromise [10,12]. These variations could be due to different PRP preparations, including or not the leukocyte fraction, which can modify its growth factor content. In both cases, PRP seemed to be a promising additive for stem cell transplantation for orthopedic applications, by increasing the number of transplanted stem cells and guiding their differentiation to a defined cell type.

The present study was proposed to establish an optimized and reproducible method for PRP preparation, and to characterize the content in growth factors and cytokines of the obtained fractions before and after platelet activation. Our final goal is to develop and characterize a PRP preparation for therapeutic purposes, focusing on high platelet yield, purity and recovery without growth factor secretion throughout sample manipulation.

## Materials and methods

### Ethics statement

All of the experimental procedures were approved by the Ethics Research Committee of the Pro-Cardiaco Hospital, Rio de Janeiro (CAAE: 04691712.3.0000.5533) and all donors signed an informed consent.

### Blood collection and platelet-rich plasma preparation

Peripheral blood from 22 healthy male and female volunteer donors (20 to 54 years old) was collected using blood collection tubes containing 0.5 ml citrate solution (Vacutainer®, Ref: 369714; BD Biosciences.

The PRP preparation procedure consisted of two centrifugation steps. All steps were performed in a refrigerated centrifuge (certified Jouan Br4i, Saint-Herblain, Loire-Atlantique, France). We studied variations in relative centrifugal force (RCF), temperature, and time for optimizing conditions for platelet isolation. After the first centrifugation, the whole plasma above the buffy coat was collected, separating platelets from red blood cells and leukocytes (PRP1). For the optimization of the second centrifugation step, only the RCF and time were studied. PRP1 from single donors was divided into 1 ml fractions and after the second centrifugation step the platelet pellet was suspended in 300 μl of platelet-poor plasma (PPP; new fraction named PRP2). PRP2 was activated using 20 mM CaCl_2_ (PRP2-Ca) or 20 mM CaCl_2_ plus 25 IU/ml human plasma thrombin (PRP2-Thr, Ref: T6884; Sigma-Aldrich, St. Louis, Missouri, USA). All samples were incubated at 37°C for 1 hour and at 4ºC for 16 hours. For recovering the activated PRP2, all of the treated samples were centrifuged at 3,000 × *g* for 20 minutes at 18°C. The supernatant (activated PRP2) was collected by aspiration. Clots were treated with 200 mM HCl for 10 minutes and neutralized with 240 mM NaOH (washed clot). All samples were stored at –80ºC.

### Hematological analysis

The total blood cell count was determined in whole blood, PRP1, PRP2 and PPP fractions. Platelet counts were performed with a hemocytometer using a Wintrobe-modified REES-ECKER fluid and other blood cell types were analyzed using a hematological analyzer XE-2100 (Sysmex, Kobe, Hyōgo, Japan).

### Cytokines quantification

Samples from peripheral blood, PRP1, PRP2, PPP, activated PRP2 and washed clot were analyzed. Proinflammatory cytokines granulocyte–macrophage colony-stimulating factor (GM-CSF), interleukin (IL)-1β, IL-6, IL-8, tumor necrosis factor (TNF)α, interferon (IFN)g, IL-2, IL-2R, IL-7, IL-12p40/p70, IL-15, IL-17), anti-inflammatory cytokines (IL-1RA, IL-4, IL-5, IL-10, IL-13, IFNα), chemokines (eotaxin, protein 10 (IP-10), monocyte chemoattractant protein-1 (MCP-1), IFNγ-induced monokine, macrophage inflammatory protein (MIP)-1α, MIP-1β, RANTES) and growth factors (endothelial growth factor (EGF), hepatocyte growth factor (HGF), basic fibroblast growth factor (bFGF), granulocyte colony-stimulating factor (G-CSF), vascular endothelial growth factor (VEGF), transforming growth factor (TGF)-β1, TGF-β2, TGF-β3, platelet-derived growth factor (PDGF)-AA, PDGF-AB, PDGF-BB, insulin-like growth factor-1 (IGF-1)) were quantified. Commercial ELISA and Luminex kits were used: Quantikine hPDGF-AB ELISA (R&D, Minneapolis, Minnesota, USA), Human Cytokine 30-plex Assay (Invitrogen, Carlsbad, California, USA), Fluorokine MAP TGF-β Multiplex Kit (R&D, Minneapolis, Minnesota, USA), Human Angiogenesis Fluorokine Multi Analyte Profiling Kit (R&D, Minneapolis, Minnesota, USA) and Milliplex MAP Human IGF-1 Single Plex Kit (Millipore, Billerica, Massachusetts, USA). Procedures were carried out following the manufacturer’s instructions.

### Statistical analysis

Results are expressed as mean ± standard deviation for at least three replicates. Statistical significance was assessed by one-way nonparametric analysis of variance followed by the Tukey *post-hoc* test (to compare all pairs in group) or Dunnett’s multiple comparison test (to compare every condition with control data but not with each other). *P* <0.05 was considered statistically significant. Statistical analysis was performed using Prism 5.00 software (GraphPad Software Inc., San Diego, California, USA).

## Results and discussion

### Optimization of the platelet-rich plasma preparation method

After initial analyses of experimental results, we decided to optimize blood processing in two centrifugation steps, described as follows. The aim of the first step was to deplete the product of red and white blood cells with minimal loss of platelets, and the aim of the second step was to obtain the highest recovery and the best yield of platelets in the smallest final plasma volume.

#### First centrifugation step

Freshly collected blood was centrifuged as described in Materials and methods, yielding three fractions: a lower dense layer containing red blood cells occupying about one-half of the total blood volume, a thin intermediate white layer containing leukocytes (buffy coat) and the upper yellow fraction containing platelets (PRP1).

The RCF, time and temperature were considered variables to be optimized, since they could potentially influence the final platelet yield and purity. These three variables are highly correlated, so one-at-a-time parameter variation would not be suitable to find the best condition because, for example, a temperature increase would reduce plasma density and therefore blood cell behavior would be different under the same centrifugation conditions; also longer centrifugation times would better combine with low RCF, so platelets are still isolated in the upper fraction.

**Figure 1:**
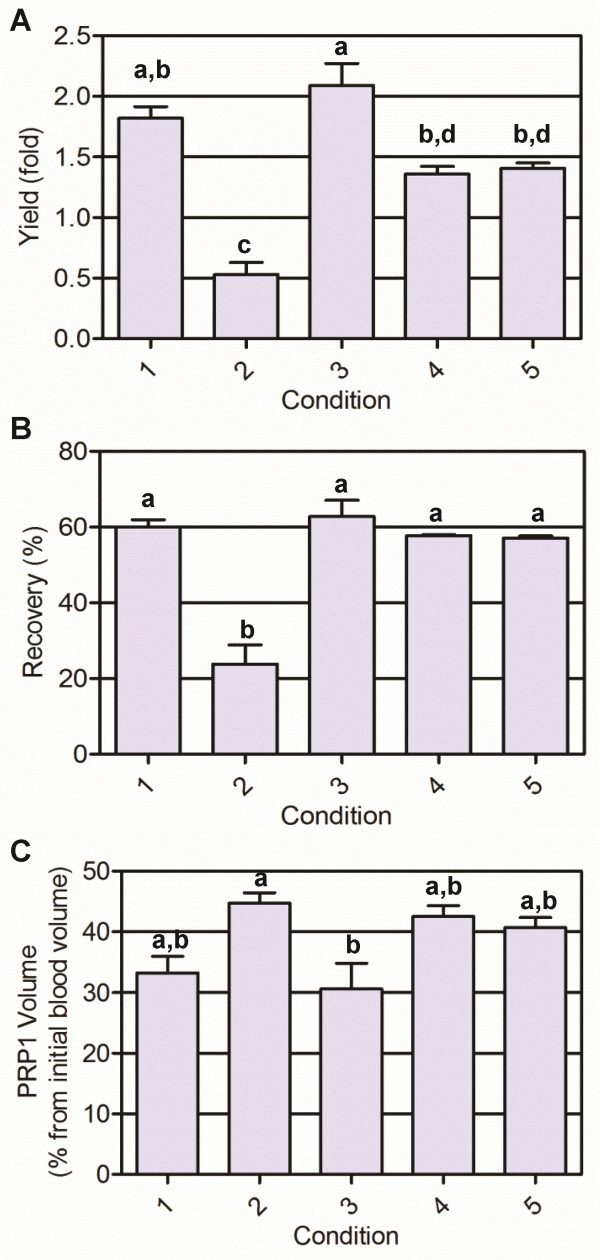
**Platelet yield, recovery and platelet-rich plasma volume after the first blood centrifugation step.** (**A**) Platelet yield, (**B**) recovery and (**C**) PRP1= platelet-rich plasma after the first blood centrifugation step. Values expressed as mean ± standard deviation. Different letters indicate statistically significant differences (analysis of variance followed by Tukey *post-hoc* test, *n* = 3, α = 0.05). Condition 1: 240 × *g*, 8 minutes, 16°C; condition 2: 360 × *g*, 16 minutes, 16°C; condition 3: 300 × *g*, 5 minutes, 12°C; condition 4: 300 × *g*, 12 minutes, 5°C; condition 5: 300 × *g*, 12 minutes, 12°C.

We ran 17 experiments, including 15 different conditions; one condition was repeated three times for error estimation. The results are presented in Table 1. We observed that increasing the RCF from 200 to 360 × *g* was detrimental for both yield and recovery. The same negative effect was observed when increasing the centrifugation time. When analyzing temperature influence, no effect was detected when centrifugation times were short; but when the centrifugation step took 16 minutes, temperature had a positive effect and increased the platelet yield. Based on these analyses, we have chosen five conditions (runs 5, 8, 11, 13 and 15) that were repeated for different blood samples. We confirmed platelet yield and recovery, showing reproducibility between days and among different donors (Figure 1). Platelet recovery and yield were statistically lower for condition 2 (360 × *g*, 16 minutes, 16°C), carried out at the highest RCF. The best performance was obtained using parameters from condition 3 (300 × *g*, 5 minutes, 12°C), the one with the lowest centrifugation time. Since a significant difference was not observed with condition 1 (240 × *g*, 8 minutes, 16°C), we chose condition 3 as a first step for further optimization of the second step, because of its shorter time.

**Table 1:** Platelet yield and recovery after total blood centrifugation

**Run**	**Parameters**			**Outputs**	
	**RCF (× *g *)**	**Centrifugation time (minutes)**	**Temperature (°C)**	**Yield (fold)**	**Recovery (%)**
1	240	8	8	2.5	66.6
2	360	8	8	0.9	30.4
3	240	16	8	1.3	52.7
4	360	16	8	0.5	19.5
5	240	8	16	2.6	69.3
6	360	8	16	1.1	33.2
7	240	16	16	1.6	10.4
8	360	16	16	1.8	73.7
9	200	12	12	2.4	82.7
10	400	12	12	0.6	24.2
11	300	5	12	5.2	87.7
12	300	19	12	0.7	28.2
13	300	12	5	1.6	62.7
14	300	12	19	0.8	30.3
15	300	12	12	1.8	73.6
16	300	12	12	1.3	53.7
17	300	12	12	1.7	69.1

*RCF* relative centrifugal force.

To monitor the platelet purity in PRP1, we quantified other cell types and we observed that erythrocytes and white blood cells were remarkably depleted from PRP1 and remained at a very low concentration (<0.3% compared with their initial concentration for both cell types; Table 2).

**Table 2:** Red blood cell and while blood cell quantification in whole blood and PRP1 fractions

**Condition**	**Volume (ml)**	**RBC (10**^**6**^**/μl)**	**WBC (10**^**3**^**/μl)**
Whole blood	4.1 ± 1.4	4.05 ± 0.28 (100%)	4.38 ± 0.47 (100%)
1 (240 × *g*, 8 minutes, 16°C)	1.7 ± 0.2	0.02 ± 0.00 (0.19%)	0.02 ± 0.02 (0.21%)
2 (360 × *g*, 16 minutes, 16°C)	2.2 ± 0.1	0.00 ± 0.00 (0%)	0.00 ± 0.00 (0.12%)
3 (300 × *g*, 5 minutes, 12°C)	1.5 ± 0.4	0.03 ± 0.01 (0.24%)	0.03 ± 0.01 (0.28%)
4 (300 × *g*, 12 minutes, 5°C)	2.0 ± 0.1	0.01 ± 0.00 (0.12%)	0.01 ± 0.00 (0.12%)
5 (300 × *g*, 12 minutes, 12°C)	2.1 ± 0.2	0.01 ± 0.00 (0.13%)	0.01 ± 0.01 (0.08%)

Values expressed as mean ± standard deviation (remaining amount of contaminant cells in PRP1, related to the initial amount and corrected by the corresponding volume). *RBC* red blood cell, *WBC* white blood cell, *PRP1* platelet-rich plasma after the first centrifugation step. *n* = 3.

#### Second centrifugation step

When optimizing the second centrifugation step, the temperature was fixed at 12°C since analyses of the first step indicated that it did not affect significantly platelet recovery and yield. Table 3 shows the results for the different conditions studied, where partial platelet yield in the platelet-rich fraction of the second run (PRP2) and partial platelet recovery in the PRP2 and the PPP were determined. In some centrifugation conditions, total platelet recovery (PRP2 plus PPP recovery values) did not reach 100%, possibly due to platelet aggregation. Important losses were observed at low RCF and the best performance was obtained using high RCF and long centrifugation times. Condition 4 (700 × *g*, 17 minutes), condition 5 (450 × *g*, 12 minutes) and condition 9 (800 × *g*, 12 minutes) where further confirmed using different blood samples (*n* = 3).

**Table 3:** Platelet yield and recovery after PRP1 centrifugation

**Run**	**Parameters**		**Yield (fold)**	**Recovery (%)**		
	**RCF (× *g *)**	**Centrifugation time (minutes)**		**PRP2**	**PPP**	**Total**
1	200	7	1.8	47.1	51.3	98.4
2	700	7	1.4	39.7	0.0	39.7
3	200	17	2.1	55.9	8.5	64.4
4	700	17	3.6	97.4	0.5	97.9
5	450	12	2.7	75.5	3.6	79.1
6	450	12	2.8	78.1	3.4	81.5
7	450	12	3.2	88.9	5.3	94.2
8	100	12	1.4	38.9	52.0	90.9
9	800	12	3.3	93.2	0.0	93.2
10	450	5	1.8	48.3	20.1	68.4
11	450	19	2.5	65.7	0.0	65.7

*PPP* platelet-poor plasma, *PRP1* platelet-rich plasma after the first blood centrifugation step, *PRP2* platelet-rich plasma after the second centrifugation step, *RCF* relative centrifugal force.

**Figure 2:**
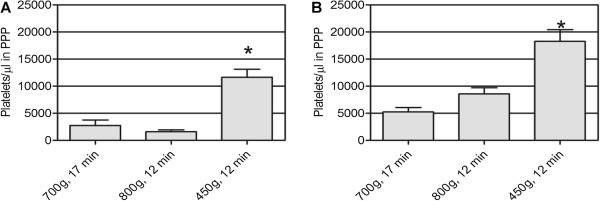
**Temperature effect on platelet loss in platelet-poor plasma fraction after the second centrifugation step.** First centrifugation was performed at the corresponding temperature: (**A**) 12°C and (**B**) 18°C. *Statistically significant differences (analysis of variance followed by Tukey *post-hoc* test, *n* = 3, α = 0.05). PPP, platelet-poor plasma.

Although the platelet recovery was not statistically different from the other two conditions *(P* = 0.3222), condition 4 (700 × *g*, 17 minutes) was chosen since it allowed a lower platelet loss into the PPP fraction (Figure 2A) and produced a pellet that was easily resuspended. For all three conditions, erythrocytes and leukocytes were quantified in both samples, PRP2 and PPP. PPP was free from erythrocytes and leukocytes. All of the remaining contaminant cells were centrifuged together with the platelets; <0.3% of the initial red and white blood cells remained in PRP2, defining our preparation as a leukocyte-poor PRP.

#### Temperature effect on platelet-rich plasma preparation method

Considering that temperature did not affect platelet isolation during the first step, the whole procedure was tested at 12°C and at 18°C (room temperature). Results are shown in Table 4 and also in Figure 2. No significant differences were observed between both temperatures.

**Table 4:** Temperature effect on platelet-rich plasma preparation method

	**12°C**	**18°C**	***P*** **value**
PRP1 (300 × *g*, 5 minutes)			
Platelet recovery (%)	90.6 ± 21.7	87.7 ± 22.8	1.0000
Platelet yield (fold)	3.1 ± 0.7	2.9 ± 0.6	0.5066
PRP1 volume (%)	26.7 ± 0.7	27.6 ± 1.9	0.7000
PRP2 (700 × *g*, 17 minutes)			
Platelet recovery (%)	80.3 ± 26.6	86.0 ± 46.9	1.0000
Platelet yield (fold)	9.3 ± 2.6	8.8 ± 3.6	0.8248

Analysis of variance test (*n* = 3, α = 0.05). *PRP1* platelet-rich plasma after the first blood centrifugation step, *PRP2* platelet-rich plasma after the second centrifugation step.

#### Method reproducibility

**Figure 3:**
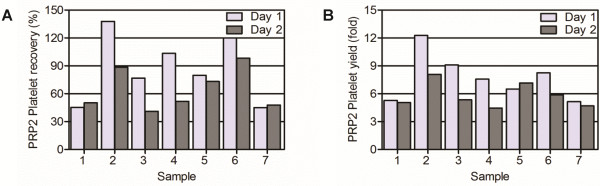
**Individual and daily variation of a platelet-rich plasma preparation at 18°C.** (**A**) PRP2 platelet recovery and (**B**) PRP2 platelet yield. Samples belong to different donors and the procedure was performed by different researchers on different days. PRP2, platelet-rich plasma volume after the second blood centrifugation step.

We tested the same procedure for different patients and on different days. The results are shown in Figure 3. For some patients (for example, Patients 1, 5 and 7) reproducibility was high, showing almost no difference between the two days.

#### Confidence interval

Considering 18 different samples and α = 0.05, our newly optimized PRP preparation method allowed us to obtain a final platelet recovery of 46.9 to 69.5%, yielding a concentration factor between 5.4 and 7.3 and a final platelet concentration of 1.4 × 10^6^ to 1.9 × 10^6^ platelets/μl.

#### Comments

In conclusion, the present study developed and characterized the following two-step method to obtain PRP, maximizing platelet purity, recovery and yield:

1) Blood samples were collected in 5 ml tubes containing 3.2% sodium citrate; a 100 μl sample was separated for determining the initial blood cell concentration.

2) Whole blood was centrifuged at 300 × *g* during 5 minutes at 18°C.

3) The upper fraction (PRP1) was separated, without disturbing the buffy coat, and was transferred into a sterile tube; the PRP1 volume was quantified and a 100 μl sample was separated for determining PRP1 platelet concentration and purity.

4) PRP1 was centrifuged at 700 × *g* during 17 minutes at 18°C.

5) PPP was transferred into a sterile tube and the platelet concentration and purity were quantified.

6) The platelet pellet obtained from 1 ml PRP1 was resuspended in 300 μl PPP (PRP2).

7) Platelet activation was performed immediately by adding 20 mM CaCl_2_ and 25 IU/ml human thrombin or CaCl_2_ alone, incubating at 37°C during 1 hour and at 4ºC during 16 hours.

8) Activated PRP2 was recovered by centrifugation at 3,000 × *g* during 20 minutes at 18°C.

We decided to collect blood in citrated glass tubes. Although Araki and colleagues recommended use of ethylenediamine tetraacetic acid to maximize platelet recovery [42], ethylenediamine tetraacetic acid use resulted in damaged platelets according to Landersberg and colleagues [43].

Anitua proposed in 1999 a simple method for obtaining PRP that has been frequently used in subsequent studies and in clinical application [44]. A fraction of 0.5 ml plasma located just above the buffy coat separated after a single centrifugation cycle (460 × *g*, 8 minutes) was collected. The upper fraction was considered PPP, since the author argued that platelet density was gradually increasing from the top to the buffy coat. In contrast, we did not find any platelet gradient in any of PRP1 fractions (data not shown), and we decided to recover the whole PRP1volume for the next centrifugation step.

The second high-speed centrifugation step concentrated platelets by one-tenth from the initial blood volume, leaving a PPP fraction with <1% platelet loss. We concentrated platelets, reaching a baseline of 1 million platelets/μl, finally recovering at least 50% of the initial platelet amount. These parameters are higher than those reported for commercial PRP kits: Sundman and colleagues reported a 1.99-fold increase in platelet concentration using an Arthrex ACP kit, minimizing the leukocyte content (0.13×); the same authors reported a 4.69-fold and 4.26-fold increase in platelet and leukocyte concentration, respectively, using a Biomet GPS III kit [45]. Mazzucco and colleagues compared four different PRP protocols: three commercially available kits (Fibrinet, RegenPRP-Kit and Plateltex) and a homemade procedure, obtaining a 1.6-fold to 4.4-fold increase in platelet concentration [29]. Le and colleagues compared a Curasan PRP kit, Plateltex, GPS II, RegenLab and a homemade protocol [30]. They obtained a 2.75-fold, 3.43-fold, 1.89-fold, 1.55-fold and 1.77-fold increase in platelet concentration and platelet recovery of 32.0%, 20.0%, 22.6%, 79.0% and 45.6%, respectively. Castillo and colleagues compared the Biomet GPS III, MTF Cascade and Arteriocyte Magellan systems and showed that platelet recovery was 22.6%, 67.6% and 65.5%, with a 2.07-fold, 1.62-fold and 2.80-fold increase in platelet concentration, respectively [31]. We obtained a high platelet recovery and a 6.4-fold increase in platelet concentration, making better use of the donor’s blood and requiring a smaller volume to obtain the same platelet amount.

Our PRP preparation is highly purified and contains almost no blood-derived cell types other than platelets. Leukocytes should be avoided in PRP preparations because of their potential proinflammatory effect [23,37,45]. Sundman and colleagues recently concluded from *in vitro* studies that leukocytes increased the catabolic PRP profile, since the catabolic cytokine concentration (matrix metalloproteinase-9 and IL-1β) was positively correlated with leukocyte concentration, but no correlation between leukocyte content and clinical effects *in vivo* has been reported to our knowledge [45].

### Growth factor and cytokine content quantification

A total of 37 growth factors and cytokines were quantified in different fractions obtained during PRP preparation: initial blood sample plasma, PRP1, PRP2, calcium-activated PRP2 (PRP2-Ca), calcium plus human thrombin-activated PRP2 (PRP2-Thr), PPP, and clot washing. Only 12 factors and cytokines were secreted from activated platelets, this being defined by statistical difference: PDGF-AA, PDGF-AB, PDGF-BB, TGF-β1, TGF-β2, EGF, IL-4, IL-8, IL-13, IL-17, IFNα and TNFα (Table 5). TGF-β3 and IFNγ were not detected in any of the fractions analyzed.

**Table 5:** Statistical comparison (q-values) of cytokine concentration between plasma and activated PRP2

**Cytokine**	**PRP2-Ca**	**PRP2-Thr**	**Result**
PDGF-AA	**6.674**	**5.931**	**Platelet-secreted factor**
PDGF-AB	**9.352**	**9.556**	**Platelet-secreted factor**
PDGF-BB	**8.072**	**7.886**	**Platelet-secreted factor**
IGF-1	0.038	0.302	Plasmatic factor
TGF-β1	**5.912**	**4.143**	**Platelet-secreted factor**
TGF-β2	**5.574**	**3.850**	**Platelet-secreted factor**
TGF-β3			Not detected
EGF	**9.243**	**8.386**	**Platelet-secreted factor**
IL-5	2.568	2.539	Plasmatic factor
IL-6	0.303	0.388	Plasmatic factor
Eotaxin	5.829	6.169	^a^
bFGF	0.101	1.173	Plasmatic factor
G-CSF	0.081	0.097	Plasmatic factor
GM-CSF	0.711	0.716	Plasmatic factor
HGF	2.657	2.469	Plasmatic factor
IFNα	**4.188**	**2.514**	**Platelet-secreted factor**
IL-1β	0.480	0.590	Plasmatic factor
IL-2	0.869	0.882	Plasmatic factor
IL-2R	0.604	0.256	Plasmatic factor
IL-4	**4.894**	**4.003**	**Platelet-secreted factor**
IL-7	1.881	1.739	Plasmatic factor
IL-1RA	1.216	0.856	Plasmatic factor
IL-8	**10.590**	**9.597**	**Platelet-secreted factor**
IL-10	1.433	1.357	Plasmatic factor
IL-12	2.079	1.070	Plasmatic factor
IL-13	**3.884**	**3.901**	**Platelet-secreted factor**
IL-15	0.001	0.337	Plasmatic factor
IL-17	**3.911**	**3.902**	**Platelet-secreted factor**
VEGF			not detected
IFNγ			not detected
IP-10	3.131	3.288	Plasmatic factor
MCP-1	4.247	4.392	^a^
MIG	1.462	1.136	Plasmatic factor
MIP-1α	0.750	1.046	Plasmatic factor
MIP-1β	3.341	2.442	Plasmatic factor
RANTES	0.463	0.839	Plasmatic factor
TNFα	**6.752**	**4.584**	**Platelet-secreted factor**

Determination of platelet-secreted cytokines using the Tukey *post-hoc* test. *q*-value = 3.68 (*k* = 3, *n* = 6, α = 0.05). *bFGF* basic fibroblast growth factor, *EGF* endothelial growth factor, *GM-CSF* granulocyte–macrophage colony-stimulating factor, *HGF* hepatocyte growth factor, *IGF-1* insulin-like growth factor-1, *IP-10* IFNγ-induced protein 10, *MCP-1* monocyte chemoattractant protein-1, *MIG* IFNγ-induced monokine, *MIP* macrophage inflammatory protein, *PDGF* platelet-derived growth factor, *PRP2* platelet-rich plasma after the second centrifugation step, *PRP2-Ca* calcium-activated PRP2, *PRP2-Thr*, calcium plus human thrombin-activated PRP2, *TGF* transforming growth factor, *VEGF* vascular endothelial growth factor. ^a^Eotaxin and MCP-1 showed statistically significant differences due to concentration reduction but they cannot be classified as platelet-secreted factors because their concentration was reduced, probably due to degradation by other products secreted following degranulation, or because they were associated with secreted molecules.

#### Growth factors

**Figure 4:**
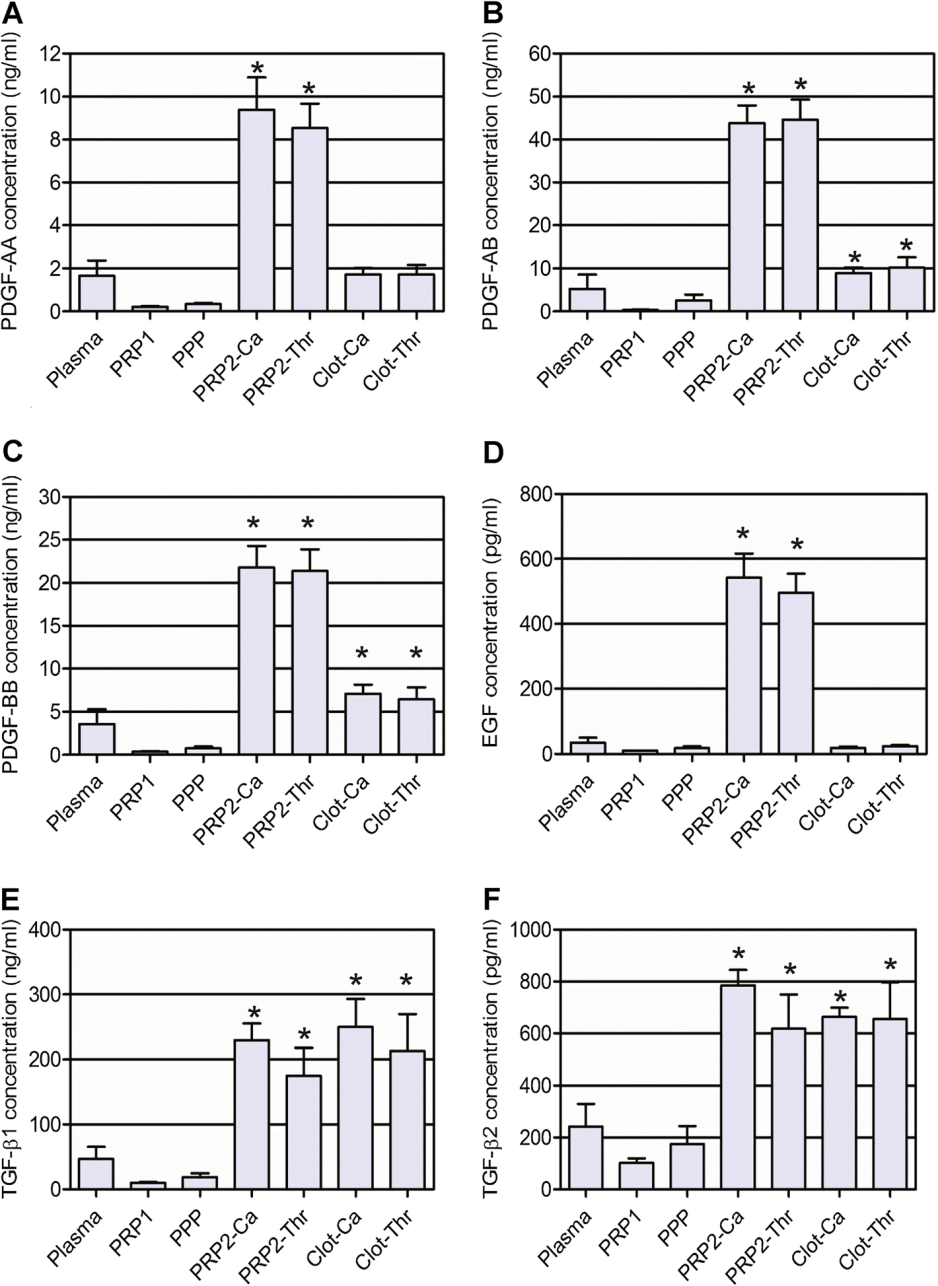
**Growth factor concentrations secreted after platelet activation. (A)** Platelet-derived growth factor (PDGF)-AA, **(B)** PDGF-AB, **(C)** PDGF-BB, **(D)** endothelial growth factor (EGF), **(E)** transforming growth factor (TGF)-β1, and **(F)** TGF-β2. *Statistically significant differences from the plasmatic concentration (analysis of variance followed by Dunnett’s multiple comparison test, *n* = 6, α = 0.05). PPP, platelet-poor plasma; PRP1, platelet-rich plasma after the first blood centrifugation step; PRP2, platelet-rich plasma after the second centrifugation step; PRP2-Ca, calcium-activated PRP2; PRP2-Thr, calcium plus human thrombin-activated PRP2.

Figure 4 shows the concentrations of growth factors secreted during platelet activation (PDGF-AA, PDGF-AB, PDGF-BB, TGF-β1, TGF-β2 and EGF). PDGF-AB and PDGF-BB showed significantly different amounts not only in activated PRP2 fractions, but also in washed clots. TGF-β1 and TGF-β2 showed significantly high growth factor concentration in PRP2-Ca but not in PRP2-Thr. The TGF clot content was the highest when compared with other growth factors. We have no information regarding the activation state of these two TGF-β isoforms because the Luminex kit quantified activated molecules so a sample processing step was performed before quantification and therefore the total TGF was detected.

To compare growth factor secretion with other publications we calculated the secreted cytokine amount per platelet, since different described protocols resulted in different platelet concentrations. Expressed by 10^6^ platelets, PRP2-Ca contained 7.9 ± 5.6 pg PDGF-AA, 37.6 ± 14.9 pg PDGF-AB, 20.1 ± 10.0 pg PDGF-BB, 318.6 ± 118.4 pg TGF-β1, 965.2 ± 389.5 fg TGF-β2 and 438.5 ± 195.3 fg EGF.

According to our criteria, growth factors already present in plasma are IGF-1, with a concentration of 105.30 ± 48.44 ng/ml (*P* = 0.9734), and hepatocyte growth factor, whose level was 61.20 ± 39.71 pg/ml (*P* = 0.1455). The plasmatic concentration did not give a significant difference with PRP2-Ca and PRP2-Thr concentrations, but they reached values 2.6-fold and 2.5-fold higher than the plasmatic level, respectively, and we consider the values indicative of secretion.

Reports on cytokines in PRP are mainly focused on the growth factors TGF, PDGF, VEGF, EGF, bFGF, HGF and IGF-1, and available quantitative data concern PDGF, TGF and EGF. Mazzocca and colleagues reported that 1,498.1 to 3,020.7 fg EGF were secreted by 10^6^ platelets, higher than the value we found [46]. Compared with our results, the same group reported a similar PDGF-AB concentration (27.4 to 48.8 pg/10^6^ platelets) and a slightly lower TGF-β1 secretion (161.7 to 185.4 pg/10^6^ platelets) [46]. Other authors reported similar values for TGF-β1: 120 pg/10^6^ platelets [47], 240 pg/10^6^ platelets [48] and 55.4 to 126.7 pg/10^6^ platelets [45]. Castilho and colleagues reported values similar to ours regarding PDGF-AB (21.9 to 44.1 pg/10^6^ platelets) and PDGF-BB (33.3 to 42.3 pg/10^6^ platelets) [31]. Reported differences reinforce the necessity of standardization of methods, in order to make the comparative clinical analyses feasible.

In our study, VEGF was detected in PRP2-Ca samples of only two out of six donors (1.93 and 1.06 pg/ml), being lower than the quantification limit of the used assay (1 pg/ml) in all of the other samples. According to the manufacturer, isoforms VEGF-121 and VEGF-165 can be detected with the assay. This is in contrast to most literature about VEGF and PRP, in which VEGF is considered one of the major factors: blood concentrations were reported to be 155 ± 110 ng/ml by Eppley and colleagues [49] and 50 ± 10 pg/ml by El-Sharkawy and colleagues [27] and PRP activation resulted in a 6.2-fold and 4.4-fold increase in VEGF concentration, respectively.

We activated PRP using calcium chloride only (20 mM), and calcium chloride plus human thrombin (25 IU/ml). We found statistically significant differences for PRP2-Ca but not for PRP2-Thr only for TGF-β1, TGF-β2 and IFNα. Even when differences were not large between the two activated fractions, these results suggested a better α-granule content secretion when platelets were activated using calcium only.

#### Anti-inflammatory cytokines

**Figure 5:**
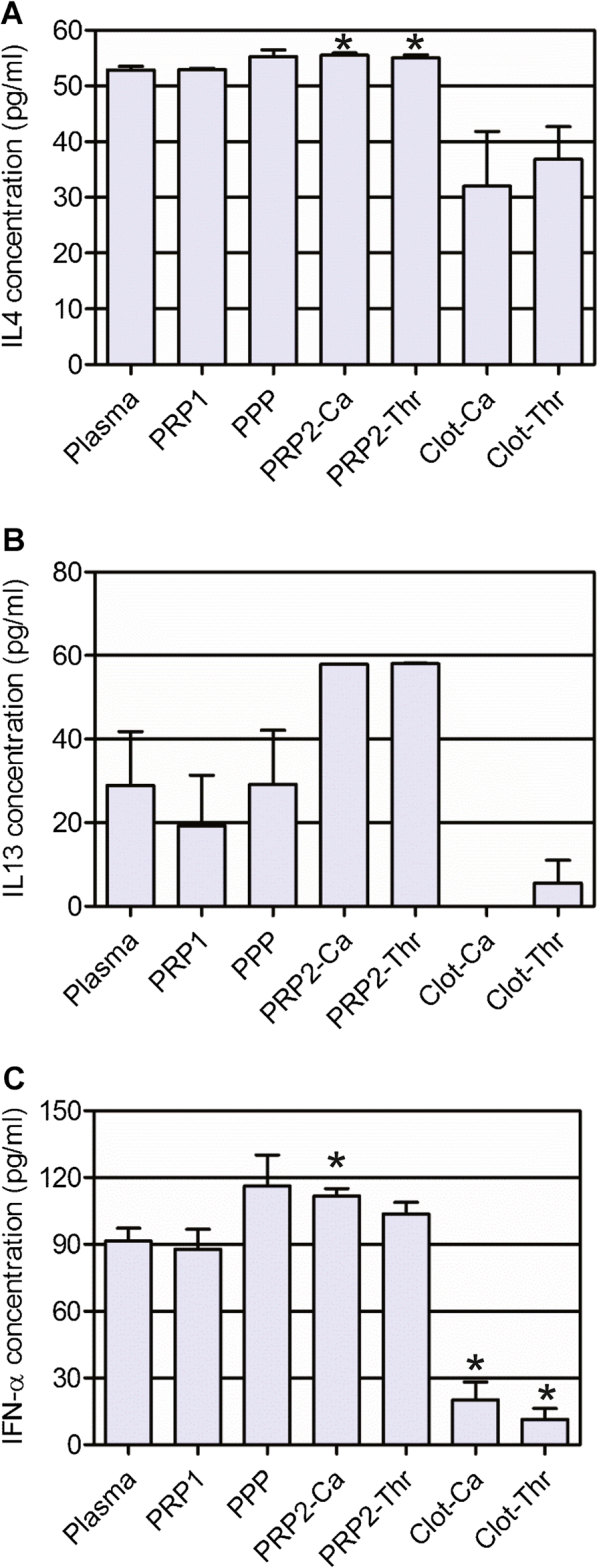
**Anti-inflammatory cytokine concentration.** Concentrations of **(A)** IL-4, **(B)** IL-13, **(C)** IFNα. *Statistically significant differences from the plasmatic concentration (analysis of variance followed by Dunnett’s multiple comparison test, *n* = 6, α = 0.05). PPP, platelet-poor plasma; PRP1, platelet-rich plasma after the first blood centrifugation step; PRP2, platelet-rich plasma after the second centrifugation step; PRP2-Ca, calcium-activated PRP2; PRP2-Thr, calcium plus human thrombin-activated PRP2.

IL-1RA, IL-5 and IL-10 showed no significant difference between activated PRP2 and plasma concentration (*P =* 0.6837, 0.1483 and 0.5361, respectively). Judging by the statistical analysis, IL-4 is an intra-platelet factor; the concentration in plasma (52.86 ± 1.63 pg/ml) and activated PRP preparations (55.53 ± 1.01 pg/ml for PRP2-Ca and 55.04 ± 1.30 pg/ml PRP2-Thr) were similar but the standard deviation was small, making differences statistically significant (*P* = 0.0079). Plasmatic IFNα (98.50 ± 13.79 pg/ml) increased by 22.09% and 13.26% in PRP2-Ca and PRP2-Thr, respectively (*P* = 0.0305). IL-13 was twice as concentrated in both activated PRP2 preparations when compared with plasma concentration (28.91 ± 31.67 pg/ml), but these differences should be considered indicative only since they were not significant (*P* = 0.0618 and 0.0509 for PRP2-Ca and PRP2-Thr, respectively). IL-4, IL-13 and IFNα concentrations are shown in Figure 5. When PRP2 was activated only with calcium chloride we obtained 34.9 ± 23.8 fg/10^6^ platelets for IFNα, 30.3 ± 28.0 fg/10^6^ platelets for IL-4 and 25.2 ± 30.4 fg/10^6^ platelets for IL-13.

#### Proinflammatory cytokines

**Figure 6:**
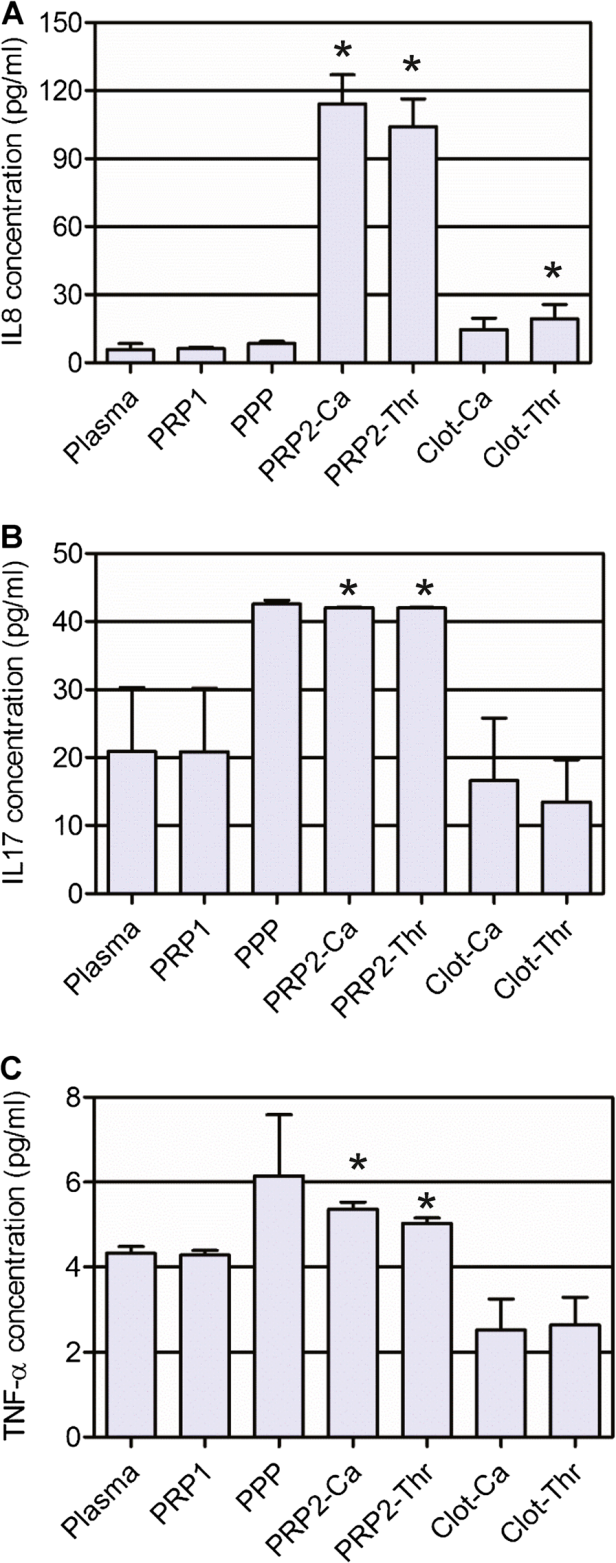
**Proinflammatory cytokine concentration.** Concentrations of **(A)** IL-8, **(B)** IL-17, and **(C)** TNFα. *Statistically significant differences from the plasmatic concentration (analysis of variance followed by Dunnett’s multiple comparison test, *n* = 6, α = 0.05). PPP, platelet-poor plasma; PRP1, platelet-rich plasma after the first blood centrifugation step; PRP2, platelet-rich plasma after the second centrifugation step; PRP2-Ca, calcium-activated PRP2; PRP2-Thr, calcium plus human thrombin-activated PRP2.

IL-17, TNFα and IL-8 were the only proinflammatory cytokines that showed a significant rise from plasma to activated PRP2 concentrations (*P* = 0.0206, 0.0008 and <0.0001, respectively). IL-1β increased its concentration 3.6 times in PRP1 and 9.5 times in PPP when compared with the plasmatic concentration (0.17 ± 0.27 pg/ml), but differences should be considered indicative since they were not statistically significant (*P* = 0.7543). The same results were obtained for granulocyte–macrophage colony-stimulating factor (*P* = 0.8456), with smaller increases (1.5-fold and 2.5-fold increase in PRP1 and PPP, respectively; plasmatic concentration: 5.48 ± 8.49 pg/ml). In the case of IL-7, there were no significant differences between plasmatic (10.60 ± 25.96 pg/ml) and activated PRP2 concentrations (*P* = 0.3592). Figure 6 shows proinflammatory cytokine concentrations for those factors secreted during platelet activation. When calculating the platelet content considering the amount of cytokine secreted during calcium activation, we obtained 98.5 ± 10.4 fg/10^6^ platelets for IL-8, 32.6 ± 24.2 fg/10^6^ platelets for IL-17, and 3.1 ± 2.4 fg/10^6^ platelets for TNFα.

While the content of growth factors was not increased in the PPP, the anti-inflammatory cytokines IL-4 and IFNα, but not IL-13, and the proinflammatory cytokines IL-17 and TNFα, but not IL-8, were increased in PPP, which is obtained after the second centrifugation. Manipulation of platelets *in vitro*, as well as sheer stress in blood vessel circulation, is known to elicit secretion of cytoplasmic mediators present in platelets into the pericellular environment, independently of their degranulation. We understand that this is the origin of the four cytokines in the PPP. Some recent reviews discuss the existence of different α-granules [50-52]. Blair and Flaumenhaft reviewed the incorporation of plasmatic proteins into the cell cytoplasm and afterwards into the α-granules, meaning that platelet membrane rupture can release factors accumulated into the cytoplasm that are also concentrated into the α-granules [50]. Ma and colleagues reported that different receptors on platelet surface play a role in regulating angiogenesis, with PAR1-mediated activation allowing platelet aggregation and VEGF secretion while suppressing endostatin release, and with PAR4-mediated activation also allowing platelet aggregation but suppressing VEGF release and stimulating endostatin secretion [53]. White and Rompietti suggested that this theory of factor segregation into different kinds of granules makes more sense than the simultaneous secretion of pro-angiogenic and anti-angiogenic factors that would cause the neutralization of the effects of the secreted proteins [52].

#### Chemokines

None of the analyzed chemokines was found to have increased content during platelet activation at a statistically significant level, but MIP-1β showed an increase from the plasmatic concentration (38.01 ± 34.23 pg/ml) when compared with PRP2-Ca (118.39 ± 82.39 pg/ml) and PRP2-Thr (96.76 ± 49.60 pg/ml) – we consider these values indicative of an increase and they should therefore be further studied. Eotaxin and MCP-1 showed significantly reduced concentration in activated PRP fractions; the eotaxin concentration dropped from a plasmatic concentration of 50.62 ± 16.95 pg/ml to 23.75 ± 8.52 pg/ml in PRP2-Ca and 22.19 ± 4.76 pg/ml in PRP2-Thr, and the MCP-1 concentration from 245.59 ± 187.52 pg/ml to 69.41 ± 43.27 pg/ml in PRP2-Ca and 78.97 ± 22.20 pg/ml in PRP2-Thr. The reduction may be caused by degradation of chemokines by the products secreted following degranulation, or their association with secreted molecules, and these issues should also be further studied.

For chemokines, only one publication for RANTES was found, reporting a plasmatic concentration of 100 ± 20 ng/mL and activated PRP concentration of 300 ± 100 ng/mL [27]. Our research resulted in plasmatic RANTES concentrations of 2.76 ± 1.97 ng/mL.

Judging by the PRP composition described here, our findings support the use of PRP in wound healing and tissue regeneration, as high concentrations of PDGF and TGF are secreted from platelet α-granules after activation. We also showed that growth factors are highly retained inside clots so, as already demonstrated by other authors, they would be slowly released *in vivo*[54,55]. These growth factors were also described as mitogenic and attractant for mesenchymal stem cells, which may continue secreting growth factors and may mediate regenerative effects during longer periods of time [56]. Our PRP preparation should now be tested *in vitro* in cell cultures in order to reveal its molecular effects on different cell types and in clinical trials in order to evaluate its *in vivo* effects in different pathologies.

## Conclusions

Our study optimized a procedure for preparing PRP from human blood, recovering more than 50% of the initial platelets with a low amount of other blood cells. This preparation was activated and different fractions collected along the procedure were analyzed in order to determine the growth factor content. Out of 37 proteins quantified, 12 were shown to be increased only after platelet activation.

## Abbreviations

bFGF: Basic fibroblast growth factor; EGF: Endothelial growth factor; ELISA: Enzyme-linked immunosorbent assay; GM-CSF: Granulocyte–macrophage colony-stimulating factor; G-CSF: Granulocyte colony-stimulating factor; HGF: Hepatocyte growth factor; IFN: Interferon; IGF-1: Insulin-like growth factor-1; IL: Interleukin; IP-10: protein 10; MCP-1: Monocyte chemoattractant protein-1; MIP: Macrophage inflammatory protein; PDGF: Platelet-derived growth factor; PPP: Platelet-poor plasma; PRP: Platelet-rich plasma; PRP1: Platelet-rich plasma after the first blood centrifugation step; PRP2: Platelet-rich plasma after the second centrifugation step; PRP2-Ca: Calcium-activated PRP2; PRP2-Thr: Calcium plus human thrombin-activated PRP2; RANTES: Regulated on activation, normal T-cell expressed and secreted; RCF: Relative centrifugal force; Thr: Human thrombin; TGF: Transforming growth factor; TNF: Tumor necrosis factor; VEGF: Vascular endothelial growth factor.

## Competing interests

The authors declare that they have no competing interests.

## Authors’ contributions

PRA, RBVC and RB participated in the design of the study. PRA, RBVC, MVTT, IdCP, and RJFCdA performed the PRP preparation. PRA, MVTT and IdCP carried out the analytical assays. PRA performed the statistical analysis. PRA and MVTT drafted the manuscript. JMG and RB revised and approved the final manuscript. All authors read and approved the final manuscript.
